# Effects of ozone therapy on postoperative pain, swelling, and trismus caused by surgical extraction of unerupted lower third molars: a double-blinded split-mouth randomized controlled trial

**DOI:** 10.4317/medoral.26974

**Published:** 2025-02-15

**Authors:** Renato Baião de Almeida, Francisco Ubiratan Ferreira de Campos, Juliana Cama Ramacciato, Daiane Cristina Peruzzo, Gustavo Vicentis Oliveira Fernandes, Julio César Joly, Marcelo Sperandio

**Affiliations:** 1 M.Sc.; Oral and Maxillofacial Rehabilitation Center, Uba, MG, Brazil; 2 M.Sc. (FUFC, JCR), Ph.D. (DCP, JCJ, MS); Faculty of Dentistry São Leopoldo Mandic, Campinas, SP, Brazil; 3 orcid: 0000-0003-3022-4390. Ph.D.; A. T. Still University - Missouri School of Dentistry and Oral Health, St. Louis, MO, U.S.A.

## Abstract

**Background:**

Third molar extraction surgery is a common procedure, but it results in pain, swelling, and trismus. Ozone therapy (Oz) has emerged as a viable option for pain control and as an option to limit bacterial growth, improving the wound healing. Then, this randomized controlled trial aimed to evaluate the effectiveness of adjunctive use of ozone therapy (OzT) in managing pain, swelling, and trismus after lower third molar removal.

**Material and Methods:**

A split-mouth design was selected, enrolling 60 patients. There were 2 groups (Sham and OzT). The same surgeon performed all procedures. The pain was evaluated using the VAS scale and the number of paracetamol Tablets taken. The quality of life was assessed using the OHIP-14 questionnaire. The data were statistically evaluated.

**Results:**

120 surgical procedures were performed on 60 participants (34 males [56%] and 26 females [44%]). Regarding the number of paracetamol Tablets taken, the test group had a significantly lower consumption (*p*<0.002). In addition, the test group presented a significantly lower pain score on days 1, 3, and 5 postoperatively, with no difference between groups on the 7th day (*p*<0.0145). Both sides presented postoperative edema, which regressed from day 5 (no significant difference). A similar case scenario was observed for mouth opening. OzT impacted the patient’s quality of life (OHIP-14, *p*<0.05), favoring ozone therapy.

**Conclusions:**

The results demonstrated that OzT is an effective adjunctive strategy for reducing postoperative pain following the extraction of lower third molar teeth.

** Key words:**Ozone therapy, lower third molar teeth, pain, swelling, trismus.

## Introduction

Third molar extraction surgery is routine in Oral and Maxillofacial Surgery (1). Generally, such teeth are fully or partially unerupted, which may cause substantial difficulty during extraction, risking trauma to bone and surrounding tissues with consequent pain, swelling, and trismus (2). Most adverse events can be attributed to inflammation from trauma and infection, the absence of which is generally considered an indirect marker of wound healing (3).

Post-operatory pain may cause substantial stress and negatively affect the quality of life. Controlling post-operative discomfort and pain following lower third molar extraction is essential to maintaining the quality of life, and many strategies have been proposed over time (4). Attempts to prevent complications and reduce recovery time using topical application of chlorhexidine, hydrogen peroxide (5), systemic corticosteroids, antibiotics (4,6), and anti-inflammatory drugs have frequently been recommended (2). However, the prescription of medication, though beneficial, increases the risk of systemic adverse reactions and toxicity, and allergic reactions (6). This fact justifies the need to investigate adjunctive strategies to manage pain, swelling, and trismus to minimize undesirable effects (7). Non-systemic methods have been proposed following third molar extraction: hyperbaric oxygen (8), cryotherapy, and laser therapy (9), even though no consensus has yet been reached on an effective protocol. Ozone therapy (Oz) has emerged as a viable option.

Oz is a gas or liquid used in a variety of ways in oral surgery, including wound healing - Oz is irrigated in extraction sites or applied as gas or ozonized oil to wounds; pain control - Oz can help with postoperative pain; antimicrobial - Oz can help limit bacterial growth; oxygen supply - Oz can increase blood flow and oxygen supply to tissues; osteonecrotic lesions - Oz can help resolve lesions that are difficult to treat with other methods (10-12). Oz is an unsTable gas with a half-life of 40 minutes at 20°C; it must be used immediately after it is created and is generated in a clinical setting using an oxygen/ozone generator that simulates lightning (13). Oz presents a characteristic odor, with high solubility and instability, easily recomposing the oxygen molecule, making it challenging to store (14). A variety of application methods can be used: gas mix (Oxygen/Ozone) for subcutaneous and intramuscular injections, liquid through ozonation of water for injection, reverse osmose or physiologic saline solution, and topical ozonized oils (15). Due to oxygen release proprieties, Oz has been used for bacterial and fungal lysis, viral inactivation, and bleeding control (16). As well as its antiseptic power and biocompatibility, Oz shows modulatory effects on inflammatory cytokines (17), growth factors release (18), and optimization of oxygen release within tissues (5). The applicability of ozone therapy (OzT) has gained considerable ground in medical and dental practices. It is expanding to many clinical situations, such as atraumatic treatment in caries control (19), root canal decontaminations (9), angiogenic stimulation in wound healing (9,14,16), and infection prevention in periodontics and implantology (9).

Amendhi *et al*. (3) performed ozone application (gaseous form) during third molar surgery and verified a decrease in the incidence of alveolitis (dry socket) postoperatively. It was attributed to the bactericidal and fungicidal effects and its modulation of the immune system. In an animal model, evidence showed that ozone application in gaseous form accelerates wound healing by increasing granulation tissue within the alveolus (15). Topical application of ozone in an oily vehicle also demonstrated a significant reduction in infection, pain, and trismus postoperatively (20). Combined application through different vehicles has seldom been explored. A possible synergistic effect needs yet to be investigated. Thus, the aim of the present study was to evaluate the adjunctive use of combined ozone as a gas and oil on pain, swelling, and trismus, as well as on the overall quality of life following surgical extraction of unerupted lower third molars.

Material and Methods

This was a randomized controlled trial (RCT) with a double-blind and split-mouth design. This study was approved by the research ethics committee of Faculdade São Leopoldo Mandic (Campinas, SP, Brazil - registration 3.270.058). This RCT was also registered on the Brazilian Clinical Trial Registry (REBEC - 9srx44).

- Study population

This study followed the Declaration of Helsinki (1975, updated 2013). It was designed following guidelines from Consort (21). Based on similar articles, sixty patients (*n*=60) were selected to participate in this study with a 14-day wash-out period. All individuals received previous information about the study and accepted and signed the informed consent form. They were referred for molar extraction and signed the informed consent form. All patients underwent a standard surgical protocol performed by the same surgeon (operator 1). Another surgeon (operator 2) conducted the ozone therapy, and a third professional (operator 3) carried out the measurements and the quality-of-life questionnaire (OHIP-14).

- Eligibility criteria

The inclusion criteria were: 1. individuals aged between 18 - 40 years; 2. with good systemic health; and 3. presenting with bilateral lower third molars, grade II-B of the Pell & Gregory’s and Winter’s classifications scale, and that needed rotatory instruments for removal. The exclusion criteria were: 1. periapical lesions, 2. signs and symptoms of pericoronitis, 3. root dilacerations, 4. smoking, 5. oral contraceptive users, and 6. pregnant and breastfeeding individuals.

- Randomization and groups

The randomization process for the patients and the first side to undergo the procedure was achieved using sealed envelopes. To avoid residual systemic effects between surgeries (8), the first tooth was extracted using sham-ozone therapy (control group), and the contralateral tooth was removed two weeks later using true ozone therapy (OzT, test group). The gas was directly applied to the socket and ozonated oil on the surgical wound.

- Surgical procedure

All patients were instructed to avoid anti-inflammatories or antibiotics 24 hours before the procedure. Local anesthesia was achieved by infiltrating three cartridges (5.4mL) of 2% lidocaine hydrochloride with 1:100.000 epinephrine (Pfizer Inc., New York, U.S.A.). The incision was followed by mucoperiosteal flap detachment and peripheral ostectomy using sterile saline-cooled rotatory instruments and cylindrical burs for access and tooth removal. The flap was repositioned, and interrupted stitches were done with nylon 5.0, followed using the control or test therapy. The contralateral third molar was removed two weeks later (wash-out), and another treatment was applied, according to the randomization.

The duration of the surgical procedure was relatively homogeneous for all patients. The post-operative pharmacological protocol included Sodium Naproxen (Akzo Nobel Polymer Chem LLC, Tennessee, U.S.A.) 500mg every 12 hours (B.D.) orally for three days and, only if required, paracetamol 750mg (Medley, Sao Paulo, Brazil) (P.R.N.), as the number of paracetamol Tablets taken had to be recorded daily. The patients were instructed to rinse with alcohol-free 0.12% chlorhexidine mouthwashes (PerioGard®, Colgate, Sao Paulo, Brazil) for 1 minute thrice daily for one week. An ice pack was applied extraorally to the surgical area for the first 30 minutes after surgery. Patients were instructed not to use other analgesics or anti-inflammatory drugs.

- Ozone and sham therapy

**Figure 1:**
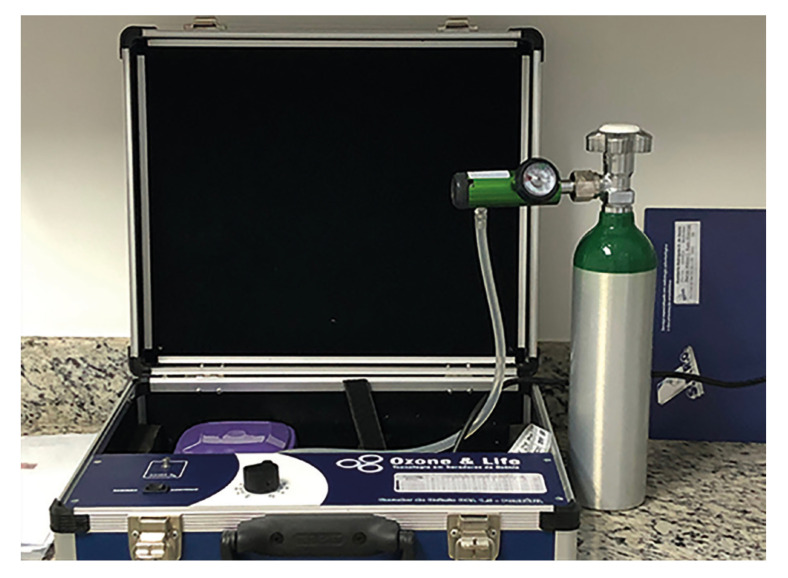
Equipment used for Ozone therapy.

Therapeutic ozone was obtained using an Ozonelife ozone generator (81509100001 ANVISA, Brazil) (Fig. 1), and the concentration of ozone selected for this study was 5 mcg/mL. The gas was generated and applied immediately to avoid concentration loss. Using a 5 mL syringe, 1 mL of ozone was applied around the surgical wound: 3 points buccally and 2 points on the lingual aspect after extraction and prior to flap suturing. It was repeated on postoperative days 1, 3, 5, and 7. Ozonated sunflower oil was applied to the sutured wound immediately after surgery and postoperatively on days 1, 3, and 5 by the same surgeon, and the sutures were removed on day 7.

The control group received the sham treatment, which consisted of using a 5 mL syringe filled with atmospheric air at the moment of therapy. One mL of gaseous ozone was gently released into the air to provide the characteristic odor associated with ozone, and the sutured surgical wound was dressed with ozone-free sunflower oil. Similar to the test group, sham-ozone application was performed on days 1, 3, and 5 (oil) and day 7 (gas).

- Postoperative data collection

The third professional measured the pain qualitatively using the visual analogical scale (VAS) on postoperative days 1, 3, 5, and 7. The participants also filled out a diary for the number of paracetamol Tablets taken until the 7th day.

Mouth opening was measured preoperatively (baseline) and postoperatively on days 1, 3, 5, and 7, as the maximal distance in millimeters between the incisal edge of the upper right central incisor (#11) and lower right central incisor (#41), using a caliper.

Edema was measured as swelling (in centimeters) using a soft tape from the auricular tragus to the labial commissure of the operated side. This was also performed at baseline and postoperatively on days 1, 3, 5, and 7. The difference between the postoperative measurements and baseline was used as a marker of edema.

Further to the aforementioned objective measurements, all patients completed a quality-of-life questionnaire (Oral Health Impact Profile - OHIP-14) (22,23) at baseline and day 7 postoperatively. It comprises 14 items that assess seven different dimensions, considering the individual's perception of the impact of oral conditions on the physical, psychological, and social well-being after procedures. Each of the 14 OHIP-14 items has a set of possible answers distributed in a Likert scale (4 = always, 3 = frequently, 2 = sometimes, 1 = seldom, and 0 = always), which represents the frequency that the individual perceives the impact of oral health on seven dimensions: functional limitation, physical pain, psychological discomfort, physical disability, psychological disability, social disability, and handicap.

- Statistical analysis

The values obtained for edema, mouth opening, number of medications taken, and pain VAS had, firstly, the normal distribution and equality of variance evaluated (Kolmogorov-Smirnov test); then, they were compared based on mean values and standard deviation (SD) using the Kruskal-Wallis test. The answers received from the OHIP-14 questionnaire (baseline and 7 days postoperatively) were compared using the Wilcoxon Test. All non-parametrical statistical calculations were performed on GraphPad Prism v.7 (GraphPad Software, LLC, California, U.S.A.) with *p*<0.05. The effect size was analyzed in order to confirm the significance found, following the interpretation for < 0.1 = trivial effect; 0.1 - 0.3 = small effect; 0.3 - 0.5 = moderate effect; and > 0.5 = large difference effect.

## Results

**Figure 2:**
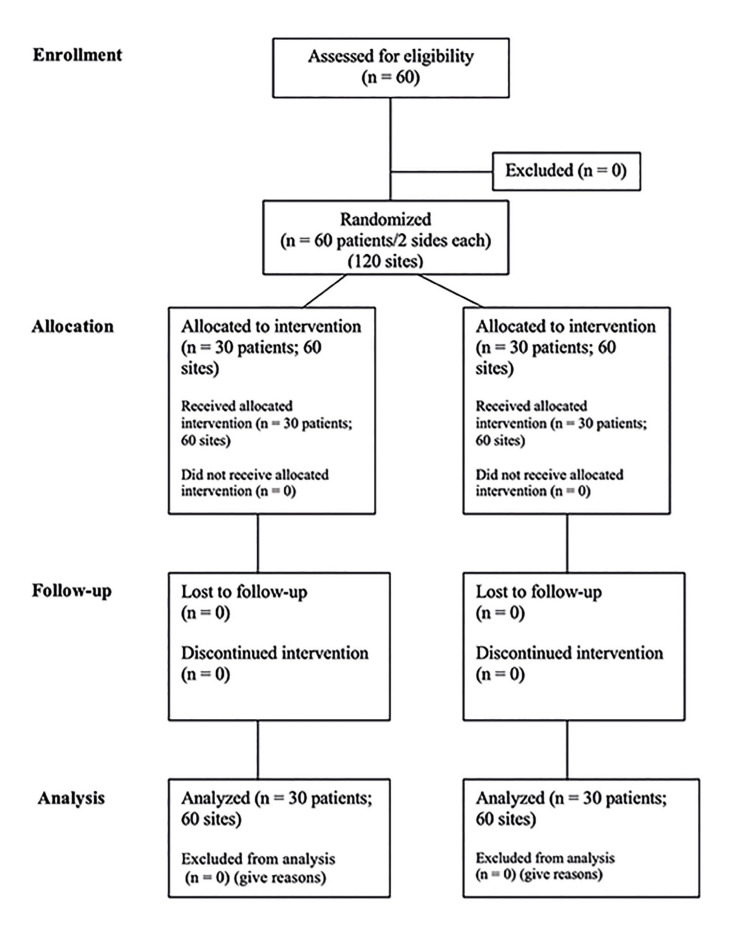
CONSORT diagram shows the flow of participants through each stage of a randomized trial.

A total of 120 surgical procedures were made in the 60 participants (34 males [56%] and 26 females [44%]), with a mean age of 25.1 ± 3.2 years (range 18 - 32 years) (Fig. 2). They were recruited between January 2020 and November 2020. All procedures were uneventful, and there was no dropout.

Regarding the number of paracetamol Tablets taken, the test group (extraction under ozone therapy) showed a significantly lower number of analgesics taken on every postoperative day evaluated (*p*<0.002, Table 1). At day 1, respectively for control and test groups, the mean and standard deviation were 3.38 ± 0.958 and 2.71 ± 0.715 (*p*=0.0001); at day 3, 2.31±0.873 and 1.85 ± 0.755 (*p*=0.0022); at day 5, 1.5 ± 0.701 and 1.0 ± 0.688 (*p*=0.0009); and at day 7, 0.73 ± 0.606 and 0.23 ± 0.464 (*p*=0.0009).

As for pain perception (VAS), a significantly lower pain score was also observed in the test group on days 1, 3, and 5 postoperatively, with no difference between groups on day 7th (*p*<0.0145, Table 2). Respectively, for control (sham O3) and test groups (true O3), on day 1, the results were 5.31 ± 1.891 and 4.76 ± 1.473 (*p*=0.0001); on day 3, 3.78 ± 1.166 and 2.85 ± 1.26 (*p*=0.0001); on day 5, 2.35 ± 1.071 and 1.7 ± 0.962 (*p*=0.0144); and day 7, 1.3 ± 0.765 and 0.93 ± 0.70 (*p*=0.03428).

**Figure 3:**
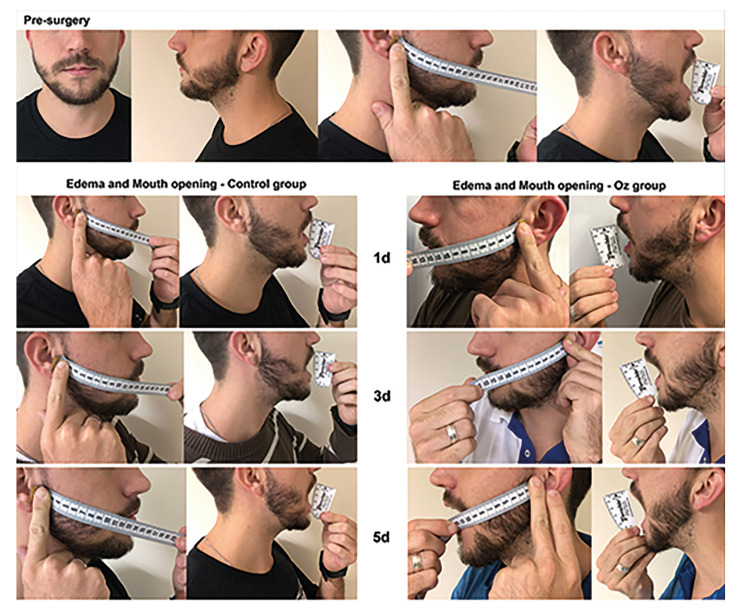
Edema and mouth opening assessment.

Both sides presented postoperative edema, especially on days 1 and 3, which regressed from day 5, with no significant difference between groups (Table 2). The results for control and test groups, respectively, were: at baseline, 12.87 ± 2.439cm and 13.57 ± 2.372cm (*p*=0.9993); at day 1, 15.03 ± 2.687cm and 14.75 ± 2.391cm (*p*=0.9764); at day 3, 14.53 ± 2.574cm and 14.28 ± 2.3cm (*p*=0.9864); at day 5, 13.7 ± 2.56cm and 13.57 ± 2.317cm (*p*=0.9993); and after 7 days, 13.0 ± 2.497cm and 13.03 ± 2.358cm (*p*>0.9999). A similar case scenario was observed for mouth opening (Table 2). The baseline values obtained were, for the control and test groups, 34.28 ± 3.45mm and 34.1 ± 3.235mm (*p*=0.9994); at day 1, 22.27 ± 3.645mm and 23.12 ± 3.966mm (*p*=0.6263); at day 3, 25.38 ± 3.547mm and 26.65 ± 3.7mm (*p*=0.2068); at day 5, 28.2 ± 3.364mm and 29.77 ± 3.088mm (*p*=0.065); and 31.58 ± 3.248mm and 32.02 ± 3.244mm (*p*=0.9664) (Fig. 3).

With regards to the impact of OzT on patient’s quality of life (OHIP-14) (Table 3), the statistics revealed a significant difference between groups (*p*<0.05), favoring the use of ozone therapy (test group) for the following questions: ‘Did you have problems pronouncing any word?’, indicating higher speech difficulty in the control group; ‘Did you feel pain in your mouth or teeth?’, indicating higher sensation of pain in the control group; ‘Did you feel worried?’, indicating a higher level of concern about their well-being in the control group, ‘Did you feel impatient/short with people around you?’, indicating a higher level of intolerance in social situations in the control group; and ‘Did you feel that your life got worse?’, indicating a generally higher perception of loss of quality of life also in the control group.

## Discussion

Even though some controlled *in vivo* studies investigating OzT are available in the literature, including its systemic administration in the gaseous form (3,7,24,25) or topical application using ozonated oil (14,20), reports remain low. The combined application (topical gas and topical oil) in a split-mouth design study, controlling a possible residual effect of the systemic OzT, has not been found in the literature insofar as the authors were aware of when writing this manuscript. However, there are discussions about the subject of OzT, raising questions about its use and the empirical and/or anecdotal data available, thereby reinforcing the necessity of high-level evidence to prove its capability.

This study was designed based on bilateral extractions of the lower third molars on a split-mouth model, testing the role of the adjunctive OzT on several markers of postoperative signs and symptoms, both objectively and subjectively. The control side was stimulated with sham-OzT (atmospheric air with ozone smell + ozone-free sunflower oil dressing), and, on the test side, the use of the true OzT after a 14-day wash-out period. Similar methodology was reported in previous studies (2-5,7,20,26,27), despite some differences in the vehicle used to carry ozone and none reporting on a combination of approaches to deliver OzT topically, as demonstrated in the present study.

The data presented herein demonstrates that OzT significantly reduced pain perception qualitatively (VAS) (2,3,5,7,20,24,26,27), reinforced by the lower number of analgesics in the test group. Such a result corroborates the findings by Kazancioglu *et al*. (7) and Sinvalingan *et al*. (6), in which the number of analgesics taken by the OzT group was also significantly lower than in the control group, even if there was the study’s design randomization for the side that received true OzT first.

When analyzing the effect of OzT on postoperative swelling and trismus, no significant difference was found between the test and control sides. This was corroborated by Kazancioglu & Kurlu (7). Conversely, Sinvalingam *et al*. (6) reported a significant reduction in edema and trismus for the OzT group (topical OzT). Therefore, the sample size was considerably smaller (*n*=33) than in the previous-mentioned study. Such methodological differences may have accounted for the contrasting outcome of the objective measurements seen between the groups among the studies discussed herein. It is notoriously difficult to quantify postoperative swelling/edema and trismus, which translates into the various methods found in the literature, such as analogic scales, images, and ultrasonography with facial parameters of references (2,4,6). In our study, postoperative swelling was measured metrically using a tape-soft tool from the auricular tragus to the labial commissure on the operated side, as recommended by Schultze-Mosgau *et al*. (20). Moreover, trismus was quantified using a linear measurement of mouth opening (mm) as an inverse marker, which was performed at baseline and repeated over the follow-up period, as recommended by Markovic and Torovic (26).

To evaluate the impact of OzT on the quality of life, the oral health impact profile questionnaire (OHIP-14) was applied at baseline (preoperative) and on day 7 postoperatively, as recommended by Kazancioglu *et al*. (7). The form was filled out manually by the participants, as suggested by Desai *et al*. (28), since the data collection route may influence the scores. Then, the participants had the freedom to respond without third-party interference. The findings revealed a favorable outcome relating to OzT compared to the control group, especially regarding speech, pain/discomfort, worries/concerns, coping, tolerance, and overall well-being. Similar to those results observed by Kazanciolgu *et al*. (7), it was possible to reinforce the overall beneficial effect of OzT practice on postoperative pain control. Considering that no dropout, complications, or adverse effects were found relating to the OzT during this study, the authors reinforce the positive reports in the literature regarding safety in its use (3,5,20).

The present study aimed to contribute to answering the possible question of systemic effect by performing the sham OzT first, thus, excluding any possible residual effect on the second intervention (15); Erdemci *et al*. (15) investigated the systemic application of ozone in an animal model study. It demonstrated that the test group benefitted significantly in alveolar bone formation postoperatively, with higher values of trabecular bone, osteoid, and osteoblasts than the control group. Such data reinforced the need to exclude a possible systemic interference of residual ozone effects on a second intervention.

The use of ozone in its original gas form immediately postoperative has been recommended (29), with the ozone concentration reducing by 50% after around 30 minutes of generating. Other authors have also described applying ozonated (test) and non-ozonated (control) oil postoperatively to dress the wound (15,20). Another differential aspect of the present study was that no systemic antibiotics were prescribed at any stage. This could risk biasing the population, especially if prescriptions were given only in the study's control arm (20).

The positive influence of OzT on postoperative symptoms and its impact on the quality of life of patients undergoing oral surgery with a potentially painful recovery period is encouraging. Moreover, topical strategies should be preferred over systemic medication (6), considering growing concerns over possible adverse effects of analgesic medication abuse.

- Study limitations

The present study had a limitation of follow-up duration which is predicTable due to the type of study performed. Moreover, we did not develop a sample size calculation because no study reported a combination of materials to do the treatment as we did. Still, we included a high level of cases/patients. Further studies with a longer duration of follow-up are suggested, including new bone formation used as a variable. Also, it is recommended to analyze the dose used herein to determine the optimum concentration and effects.

## Conclusions

Within the limitation of this study, our findings demonstrated that adjunctive OzT was an effective and safe strategy to reduce pain and analgesic intake postoperatively, positively impacting the overall quality of life.

## Figures and Tables

**Table 1:** Mean and standard deviation of number of paracetamol tablets taken postoperatively.

Group/ *p-value*/effect	Day 1	Day 3	Day 5	Day 7
Control group (Sham O3)	3.38 ± 0.958	2.31 ± 0.873	1.5 ± 0.701	0.73 ± 0.606
Test group (True O3)	2.71 ± 0.715	1.85 ± 0.755	1.0 ± 0.688	0.23 ± 0.464
P-value	0.0001	0.0022	0.0009	0.0009
Effect size analysis	0.6993737****	0.52691867****	0.71326676****	0.82508251****

**** = large difference effect.

**Table 2:** Mean and standard deviation: A. Pain score (VAS) postoperatively; B. Swelling (cm) at baseline and during follow-up.; C. Mouth opening (mm) at baseline and during follow-up.

Group/*P-value*/effect		Baseline	Day 1	Day 3	Day 5	Day 7
A. Pain score	Control group (Sham O3)	-	5.31 ± 1.891	3.78 ± 1.166	2.35 ± 1.071	1.3 ± 0.765
	Test group (True O3)	-	4.76 ± 1.473	2.85 ± 1.26	1.7 ± 0.962	0.93 ± 0.70
	*P-value*	-	0.0001	0.0001	0.0144	0.03428
	Effect size analysis	-	0.2908514**	0.79759863****	0.60690943****	0.48366013***
B. Swelling (cm)	Control group (Sham O3)	12.87 ± 2.439	15.03 ± 2.687	14.53 ± 2.574	13.7 ± 2.56	13.0 ± 2.497
	Test group (True O3)	13.57 ± 2.372	14.75 ± 2.391	14.28 ± 2.3	13.57 ± 2.317	13.03 ± 2.358
	*P-value*	0.9993 (NS)	0.9764 (NS)	0.9864 (NS)	0.9993 (NS)	>0.9999 (NS)
	Effect size analysis	0.2870029**	0.10420543**	0.0971251*	0.05078125*	0.0120144*
C. Mouth opening (mm)	Control group (Sham O3)	34.28 ± 3.45	22.27 ± 3.645	25.38 ± 3.547	28.2 ± 3.364	31.58 ± 3.248
	Test group (True O3)	34.1± 3.235	23.12 ± 3.966	26.65 ± 3.7	29.77 ± 3.088	32.02 ± 3.244
	*P-value*	0.9994 (NS)	0.6263 (NS)	0.2068 (NS)	0.065 (NS)	0.9664 (NS)
	Effect size analysis	0.05217391*	0.2331962**	0.3580491***	0.4667063***	0.135468**

NS = non-significant; * = trivial effect; ** = small effect; *** = moderate effect; **** = large difference effect.

**Table 3:** OHIP-14 questionnaire and the results obtained.

OHIP-14 Questionnaire	p-value	Significant results (p<0.05)
1. Have you ever had difficulty pronouncing words/ sentences because of problems with your oral cavity?	0.0156	yes
2. Have you ever felt unable to taste well because of problems with your oral cavity?	0.0013	no
3. Have you ever had pain in your mouth?	0.0213	yes
4. Have you ever felt uncomfortable when chewing because of problems in the oral cavity?	0.0649	no
5. Have you ever felt worried/anxious because of problems with your oral cavity?	0.0312	yes
6. Have you ever felt tense because of problems with your oral cavity	0.0005	no
7. Have you ever felt dissatisfied with the food your consumed because of problems with your oral cavity?	0.0039	no
8. Have ever had to stop suddenly while chewing food because of problems in the oral cavity?	0.0520	no
9. Have you ever had difficulty feeling relaxed because of problems in the oral cavity?	0.0005	no
10. Have you ever felt embarrassed because of problems with your oral cavity?	0.002	no
11. Have you ever become irritable because of problems in the oral cavity?	0.0391	yes
12. Have you ever had difficulty carrying out your daily activities because of problems with your oral cavity?	<0.0001	no
13. Have you ever felt that your life is unsatisfactory because of problems with your oral cavity?	0.0312	yes
14. Have you ever found it difficult to do anything because of oral problems?	0.002	no

## References

[1] Uzeda MJ, Silva AMO, Costa LN, Brito FS, Fernandes GVO, Resende RF (2024). Evaluating the effectiveness of low-level laser therapy in patients undergoing lower third molar extraction: A double-blinded randomized controlled trial. Med Oral Patol Oral Cir Bucal.

[2] Mojsa IM, Pokrowiecki R, Lipczynski K, Czerwonka D, Szczeklik K, Zaleska M (2017). Effect of submucosal dexamethasone injection on postoperative pain, oedema, and trismus following mandibular third molar surgery: a prospective, randomized, double bind clinical trial. J Oral Maxillofac Surg.

[3] Ahmendi J, Ahmendi E, Sejfija O, Agani Z, Hamiti V (2016). Efficiency of gaseous ozone in reducing the development of dry socket following surgical third molar extraction. Eur J Dent.

[4] Grossi GB, Maiorana C, Garramone RA, Borgonovo A, Creminelli L, Santoro F (2007). Assessing postoperative discomfort after third molar surgery: a prospective study. J Oral Maxillofac Surg.

[5] Mckenna DF, Borzabadi-Farahani A, Lynch E (2013). The Effect of Subgingival Ozone and/or Hydrogen Peroxide on the Development of Peri-implant Mucositis: A Double-Blind Randomized Controlled Trial. Int J Oral Maxillofac Imp.

[6] Sivalingam VP, Panneerselvam E, Krishnakumar VBR, Gopi G (2017). Does topical ozone therapy improve patient comfort after surgical removal of impacted mandibular third molar? randomized controlled trial. J Oral Maxillofac Surg.

[7] Kazancioglu HO, Kurlu E, Ezirganli S (2014). Effects of ozone therapy on pain, swelling, and trismus following third molar surgery. Int J Oral Maxillofac Surg.

[8] Kan B, Sencimen M, Bayar GR, Korkusuz P, Coskun AT, Korkmaz A (2015). Histomorphometric and Microtomographic evaluation of the effects of hyperbaric oxygen and systemic ozone, used alone and in combination, on calvarial defect healing in rats. J Oral Maxillofac Surg.

[9] John SS, Mohanty S, Chaudhary Z, Sharma P, Kumari S, Verma A (2020). Comparative evaluation of Low Level Laser Therapy and cryotherapy in pain control and wound healing following orthodontic tooth extraction: A double blind study. J Craniomaxillofac Surg.

[10] Satapathy A, Balani A, Kharsan V, Karan A, Mazhar H, Awasthy A (2023). Topical-Ozonized Olive Oil - A Boon for Post-Extraction Cases: A Randomized Controlled Trial. Cureus.

[11] Domb WC (2014). A Brief Review for Physicians. Ozone Therapy in Dentistry. Interv Neuroradiol.

[12] El Meligy OA, Elemam NM, Talaat IM (2023). Ozone Therapy in Medicine and Dentistry: A Review of the Literature. Dent J.

[13] Saini R (2011). Ozone therapy in dentistry: A strategic review. J Nat Sci Biol Med.

[14] Wentworth Jr P, McDunn JE, Wentworth AD, Takeuchi C, Nieva J, Jones T (2002). Evidence for Antibody-Catalyzed Ozone Formation in Bacterial Killing and Inflammation. Science.

[15] Erdemci F, Gunaydin Y, Sencimen M, Bassorgun I, Ozler S, Gulses A (2014). Histomorphometric evaluation of the effect of systemic and topical ozone on alveolar bone healing following tooth extraction in rats. J Oral Maxillofac Surg.

[16] Borges GA, Elias ST, Silva SMM, Magalhaes PO, Macedo SB, Ribeiro APD (2017). In vitro evaluation of wound healing and antimicrobial potential of ozone therapy. J Craniomaxillofac Surg.

[17] Sire A, Marotta N, Ferrillo M, Agostini F, Sconza C, Lippi L (2022). Oxygen-Ozone Therapy for Reducing Pro-Inflammatory Cytokines Serum Levels in Musculoskeletal and Temporomandibular Disorders: A Comprehensive Review. Int J Mol Sci.

[18] Slade GD, Foy SP, Shugars DA, Phillips C, White Jr RP (2004). The impact of third molar symptoms, pain, and swelling on oral health-related quality of life. J Oral Maxillofac Surg.

[19] Duggal MS, Nikolopoulou A, Tahmassebi IF (2012). The additional effect of ozone in combination with adjunct remineralisation products on inhibition of demineralization of the dental hard tissues in situ. J Dent.

[20] Schultze-Mosgau S, Schmelzeisen R, Frolich JC, Schmele H (1995). Use of Ibuprofen and Methylprednisolone for the Prevention of Pain and Swelling After Removal of Impacted Third Molar. J Oral Maxillofac Surg.

[21] Moher D, Schulz KF, Gøtzsche PC, Egger M (2010). CONSORT 2010 Explanation and Elaboration: updated guidelines for reporting parallel group randomised trials. BMJ.

[22] Slade GD, Spencer AJ (1994). Development and evaluation of the oral health impact profile. Community Dent Health.

[23] Campos LA, Peltomäki T, Marôco J, Campos JADB (2021). Use of Oral Health Impact Profile-14 (OHIP-14) in Different Contexts. What Is Being Measured? Int J Environment Res Public Health.

[24] Doğan M, Doğna OD, Düger C, Kol IO, Akpinar A, Mutaf B (2014). Effects of High-Frequency Bio-Oxidative Ozone Therapy in Temporomandibular Disorder-Related Pain. Med Princ Pract.

[25] Isler SC, Unsal B, Soysal F, Ozcan G, Peker E, Karaca IR (2018). The effects of ozone therapy as an adjunct to the surgical treatment of peri-implantitis. J Periodontal Implant Sci.

[26] Marković AB, Todorović L (2006). Postoperative analgesia after third molar surgery: contribution of the use of long-acting local anesthetics, low-power laser and diclofenac. Oral Surg Oral Med Oral Pathol Oral Radiol Endod.

[27] Porto GG, Vasconcelos BCE, Gomes ACA, Albert D (2007). Evaluation of lidocaine and mepivacaine for inferior third molar surgery. Med Oral Patol Oral Cir Buccal.

[28] Desai R, Durham J, Wassel RW, Preshaw PM (2014). Does the mode of administration of the Oral Health Impact Profile-49 affect the outcome score?. J Dent.

[29] Barczyk I, Masłyk D, Walczuk N, Kijak K, Skomro P, Gronwald H (2023). Potential Clinical Applications of Ozone Therapy in Dental Specialties-A Literature Review, Supported by Own Observations. Int J Environ Res Public Health.

